# Adulteration Detection of Multi-Species Vegetable Oils in Camellia Oil Using SICRIT-HRMS and Machine Learning Methods

**DOI:** 10.3390/foods15030434

**Published:** 2026-01-24

**Authors:** Mei Wang, Ting Liu, Han Liao, Xian-Biao Liu, Qi Zou, Hao-Cheng Liu, Xiao-Yin Wang

**Affiliations:** 1 Ganzhou General Inspection and Testing Institute, China National Quality and Inspection Center for Se-Rich and Camellia Oleifera Products (Jiangxi), Ganzhou 341000, China; wangmei84@163.com (M.W.); tliu0221@163.com (T.L.); liaohan871109@163.com (H.L.); 18370813589@163.com (X.-B.L.); 2 School of Public Health and Health Management, Gannan Medical University, Ganzhou 341000, China; ansishc@163.com; 3 Key Laboratory of Development and Utilization of Gannan Characteristic Food Function Component of Ganzhou, Gannan Medical University, Ganzhou 341000, China

**Keywords:** camellia oil, adulteration detection, SICRIT-HRMS, machine learning

## Abstract

We aimed to establish a rapid and precise method for identifying and quantifying multi-species vegetable oil (corn oil, olive oil (OLO), soybean oil, and sunflower oil (SUO)) adulterations in camellia oil (CAO), using soft ionization by chemical reaction in transfer–high-resolution mass spectrometry (SICRIT-HRMS) and machine learning methods. The results showed that SICRIT-HRMS could effectively characterize the volatile profiles of pure and adulterated CAO samples, including binary, ternary, quaternary, and quinary adulteration systems. The low *m*/*z* region (especially 100–300) exhibited importance to oil classification in multiple feature-selection methods. For qualitative detection, binary classification models based on convolutional neural networks (CNN), Random Forest (RF), and gradient boosting trees (GBT) algorithms showed high accuracies (98.70–100.00%) for identifying CAO adulteration under no dimensionality reduction (NON), principal component analysis (PCA), and uniform manifold approximation and projection (UMAP) strategies. The RF algorithm exhibited relatively high accuracy (96.25–99.45%) in multiclass classification. Moreover, the five models, including CNN, RF, support vector machines (SVM), logistic regression (LR), and GBT, exhibited different performances in distinguishing pure and adulterated CAO. Among 1093 blind oil samples, under NON, PCA, and UMAP: 10, 5, and 67 samples were misclassified by CNN model; 6, 7, and 41 samples were misclassified by RF model; 8, 9, and 82 samples were misclassified by SVM model; 17, 18, and 78 samples were misclassified by LR model; 7, 9, and 43 samples were misclassified by GBT model. For quantitative prediction, the PCA-CNN model performed optimally in predicting adulteration levels in CAO, especially with respect to OLO and SUO, exhibiting a high coefficient of determination for calibration ($R_{C}^{2}$, 0.9664–0.9974) and coefficient of determination for prediction ($R_{p}^{2}$, 0.9599–0.9963) values, low root mean square error of calibration (RMSEC, 0.9–5.3%) and root mean square error of prediction (RMSEP, 1.1–5.8%) values, and RPD (5.0–16.3) values greater than 3.0. These results indicate that SICRIT-HRMS combined with machine learning can rapidly and accurately identify and quantify multi-species vegetable oil adulterations in CAO, which provides a reference for developing non-targeted and high-throughput detection methods in edible oil authenticity.

## 1. Introduction

Camellia oil (CAO), extracted from the seeds of *Camellia oleifera* Abel, is rich in unsaturated fatty acids, with a content as high as 85–97% [1]. It is often termed “eastern olive oil” as its fatty acid composition is similar to that of olive oil [2]. It is a high-quality edible oil with high medicinal and nutritional value, as it also contains a multitude of bioactive components such as sterol, squalene, polyphenols, sasanquasaponin, tocopherols, γ-tocopherol, and so on [1]. It has a variety of pharmacological effects and biological activities, such as hypolipidemic, neuroprotective, skin repair, anti-obesity, antioxidant, anti-inflammatory, antimicrobial, hepatoprotective, and anti-tumor activities [3]. These properties make CAO popular among consumers and give it a higher market price than other vegetable oils. To make it more economically valuable, CAO is often adulterated with cheaper vegetable oils, such as corn oil (COO), sunflower oil (SUO), soybean oil (SOO), and olive oil (OLO) [4]. This practice undermines CAO’s unique quality and nutritional value, and can even destabilize local CAO market economies. Therefore, it is crucial to detect and prevent such adulterations to guarantee the orderly development of the CAO industry and market health.

Various analytical methods have been designed and used to detect CAO authentication, most of which are chromatographic [5,6,7,8] and spectroscopic [2,9,10,11,12,13,14,15] techniques and their combinations [16]. In terms of chromatographic techniques, characteristic volatile component gas chromatography–mass spectrometry (GC-MS) fingerprints combined with chemometrics have successfully been used for CAO grading adulteration detection [8]. Detection of CAO adulteration with palm superolein, refined OLO, high oleic-SUO, SUO, COO, rice bran oil, rice oil, peanut oil, sesame oil, SOO, and rapeseed oil has been performed using chemometrics based on fatty acid GC fingerprints and phytosterol GC-MS fingerprints [5]. Multiplex camellia oil adulteration with COO, SUO, SOO, and rapeseed oil has been identified and quantified based on 11 characteristic lipids using ultra-performance liquid chromatography (UPLC)-Q-Orbitrap-MS [6]. Multiplex CAO adulteration with COO, SUO, SOO, and peanut oil has been identified based on lipidomic fingerprints using laser-assisted rapid evaporative ionization mass spectrometry [7]. Regarding spectroscopic techniques, proton nuclear magnetic resonance [2], near-infrared spectroscopy [9,11,15,17,18], Fourier-transform infrared spectroscopy [12], Raman spectroscopy [10], Surface-enhanced Raman spectroscopy [14], and excitation–emission matrix fluorescence spectroscopy [13] have been used to detect CAO adulteration with COO, SUO, SOO, OLO, rapeseed oil, peanut oil, sesame oil, palm oil, rice oil, linseed oil, and/or maize oil. Although widely used for detecting CAO adulteration, chromatographic and spectroscopic techniques have limitations. For instance, chromatographic techniques have been reported to be tedious and destructive, and they often require complicated sample pretreatment [2,4]. Spectroscopic techniques are expensive and require time-consuming data analysis, generate only spatial information, require a reference technique for the calibration of equipment in every instance, cannot identify the individual chemical properties of the adulterants present, etc. [4,19]. Therefore, further efforts to discover novel techniques for CAO adulteration detection are needed. Yet, current studies mainly focus on binary adulteration detection (CAO and one other oil), and very few methods for detecting multi-species vegetable oil adulterations in CAO have been developed. Since it is more difficult to achieve precise and effective results with multi-species vegetable oil detection relative to binary adulteration detection [15], establishing novel methods for identifying and quantifying the adulteration of multi-species vegetable oils in CAO is of great significance for guaranteeing the orderly development of the CAO industry and market health.

Ambient ionization mass spectrometry (AIMS) has attracted significant attention in the field of food analysis due to its remarkable ability to analyze solid, liquid, and gas samples with simplicity and high throughput [20]. AIMS has been employed in determining food adulteration because it allows for fast and convenient analysis [21]. For example, AIMS with principal component analysis has been employed for the rapid characterization and classification of edible oils, in which non-edible and gutter oils could be clearly distinguished from edible oils [22]. Soft ionization by chemical reaction in transfer (SICRIT) is an ambient ionization technique based on flowing dielectric barrier discharge. It enables direct, high-sensitivity, and wide-coverage mass spectrometric detection of volatile organic compounds and semi-volatile substances without the need for sample pretreatment or the use of solvents or auxiliary gases. High-resolution mass spectrometry (HRMS) is a reliable tool for determining food authenticity, as HRMS detectors offer advantages in terms of their resolving power, sensitivity, robustness, extended dynamic range, easier mass calibration, and tandem mass capabilities [23]. HRMS has been used to detect whether OLO has been adulterated with SUO, SOO, hazelnut oil, and avocado oil [24], as well as with soft-refined oils [25]. Furthermore, the SICRIT-HRMS method might be a greener and faster alternative for food quality assessment relative to conventional chromatographic techniques [26]. Moreover, it has successfully been used for detailed chemical analysis of a fully formulated oil [27]. Therefore, based on the above background, the SICRIT-HRMS method might be a promising new approach for identifying CAO adulteration.

CAO adulteration (especially multi-component adulteration) detection usually involves a time-consuming data-processing stage. Machine learning, an advanced data analysis technique, provides a promising strategy for the automated data analysis of perplexing relationships between various samples [7]. Combinations of machine learning and spectroscopic techniques, including Raman spectroscopy [10], Surface-enhanced Raman spectroscopy [14], near-infrared spectroscopy [11,15,18], and laser-assisted rapid evaporative ionization mass spectrometry [7], have been used to efficiently identify various forms of CAO adulteration. Moreover, deep learning models have outperformed chemometric methods in quantitatively predicting the adulteration level of CAO [10]. Furthermore, SICRIT-HRMS and machine learning have been combined for the assessment of production waste samples from a dismantled synthetic drug laboratory [28]. Thus, combining SICRIT-HRMS and machine learning might be a suitable route for detecting CAO adulteration.

Hence, in this study, combinations of SICRIT-HRMS and machine learning methods were developed for identifying and quantifying multi-species vegetable oil (COO, OLO, SOO, and SUO) adulteration in CAO. A SICRIT-HRMS method combined with a volatile metabolomics approach was employed to capture fingerprints of CAO along with its adulterated oils. Moreover, machine learning models, including convolutional neural networks (CNN), Random Forest (RF), support vector machines (SVM), logistic regression (LR), and gradient boosting trees (GBT) models, were constructed for qualitative and classification detection of adulterated CAO. Furthermore, the CNN model was developed to quantitatively predict adulteration levels in adulterated CAO. This study establishes a non-targeted, rapid, and highly sensitive identification technology for CAO adulteration, which is expected to provide ideas and references for developing the authenticity identification method of CAO.

## 2. Materials and Methods

### 2.1. Samples and Preparation

CAO samples (*n* = 107), originating from the Jiangxi, Guangxi, and Hunan Provinces (three major CAO production regions in China), were obtained via hydraulic cold pressing in our laboratory or purchased from CAO production enterprises. Four other types of commercially available refined vegetable oils, including COO (*n* = 15, from twelve different brands), OLO (*n* = 15, from fourteen different brands), SOO (*n* = 15, from nine different brands), and SUO (*n* = 15, from twelve different brands), were bought from JD.com. Information regarding the samples of these five vegetable oils is listed in Table S1 (available in the Supplementary Materials).

To establish adaptable models, adulterated CAO samples with more extensive information were prepared. Specifically, thirty CAO samples were selected from among the above-mentioned CAO samples and then mixed to prepare a blended CAO sample. Meanwhile, COO, OLO, SOO, and SUO from different brands were mixed to prepare blended COO, OLO, SOO, and SUO samples, respectively. Finally, these blended CAO, COO, OLO, SOO, and SUO samples were used to prepare binary, ternary, quaternary, and quinary adulteration systems. In the binary adulteration system, the blended COO, OLO, SOO, or SUO samples were added to the blended CAO sample in percentages ranging from 3% to 90% (3%, 5%, 10%, 20%, 30%, 40%, 50%, 60%, 70%, 80%, and 90%, *v*/*v*). In the ternary adulteration system, the blended COO, OLO, SOO, and SUO samples were first mixed in pairs at a volume ratio of 1:1, and then the mixtures were added into the blended CAO sample in the same percentages. For the quaternary adulteration system, combinations of three of the blended COO, OLO, SOO, and SUO samples were first mixed at a volume ratio of 1:1:1, and then the mixtures were added into the blended CAO sample in the same percentages. In the quinary adulteration system, the blended COO, OLO, SOO, and SUO samples were first mixed in pairs at a volume ratio of 1:1:1:1, and then the mixtures were added to the blended CAO sample at the same percentages. Each adulterated gradient had three replicates, and the volume of each adulterated CAO sample was 10 mL. In total, 165, 198, 132, and 33 adulterated CAO samples were prepared in the four adulteration systems noted above, respectively. All the samples were subjected to SICRIT-HRMS measurement.

### 2.2. SICRIT-HRMS Measurement

Adulterated CAO samples were detected under positive-ion mode using an SICRIT-HRMS system consisting of an SICRIT SC-30X ion source (Plasmion GmbH, Augsburg, Germany) equipped with a Thermo Scientific Orbitrap Exploris 120 MS (Waltham, MA, USA), according to the study of Basham et al. [27] with slight modifications. Samples (2.0 mL) were placed in a 5 mL centrifuge tube and incubated at 50 °C for 5 min in a water bath. Then, the centrifuge tube was placed at the inlet end of the SICRIT ion source for 5 s to collect MS data. The operating conditions of the SICRIT ion source were as follows: a collection voltage of 1.5 kV and a frequency of 15 kHz. MS acquisition was operated using Full MS/data-dependent MS^2^ (Full MS/dd-MS^2^) mode acquisition in a scan range of *m*/*z* 75–1000. The instrument’s resolution was set to 60,000 full width at half-maximum (FWHM) for full MS scans and 15,000 FWHM for MS^2^ scans. The MS operating conditions were the following: a spray voltage, sheath gas flow rate, and auxiliary gas flow rate of zero; a capillary temperature of 320 °C; and experiments were performed with dynamic exclusion to avoid unnecessary MS/MS information acquisition.

### 2.3. Data Preprocessing

**Figure 1:**
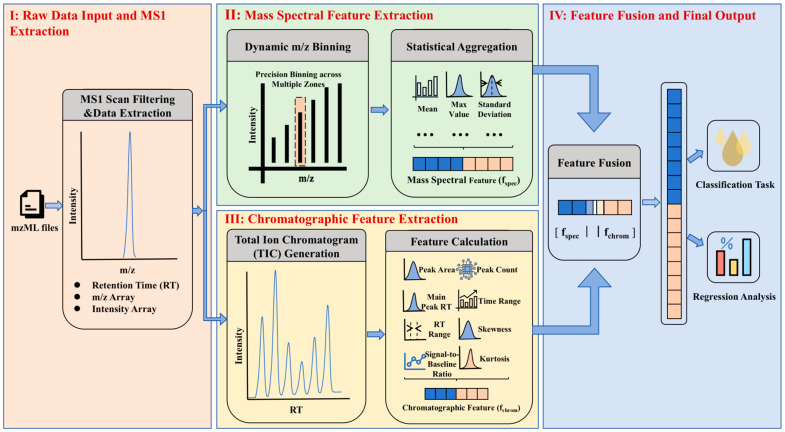
Schematic workflow of the SICRIT-MS data processing pipeline.

Raw MS files were converted into mzXML format using MSConvert from the Proteowizard Suite (proteowizard.sourceforge.net). Subsequent data processing and machine learning model training/testing were performed in Python (version 3.6.8). A schematic workflow of the data-processing pipeline is provided in Figure 1. The raw mass spectrometry data (*m*/*z* 75–1000) were first subjected to dynamic binning across 92 predefined intervals based on characteristic lipid compounds in edible oils. Each interval, defined by a start value, an end value, and a step size (e.g., 0.01 Da in the interval 74.99–75.11), was divided into equal sub-bins according to the specified step size. Signal intensities within each sub-bin were summed, yielding a total of 666 binned features. These features were then aggregated along the time axis using five statistical parameters (maximum, mean, median, standard deviation, and sum), resulting in a feature matrix with 666 × 5 = 3330 dimensions. Although SICRIT-HRMS is an ambient ionization technique, a total ion chromatogram (TIC) was still constructed from the time-series scanning data. Eight chromatographic features were extracted from the TIC, including the total peak area, the retention time of the main peak, the signal-to-noise ratio, the number of peaks, and shape descriptors (skewness and kurtosis). The final feature vector was obtained by concatenating the time-aggregated binned features (3330) with the chromatographic features (8), resulting in a total dimension of 3338. After feature augmentation and data shuffling, the dataset was split into training and test sets (8:2 ratio).

Moreover, two distinct dimensionality reduction techniques, principal component analysis (PCA) and uniform manifold approximation and projection (UMAP), were implemented to enhance computational efficiency and optimize feature representation. The resulting reduced-dimension features were systematically stored as independent datasets, thereby establishing three comparative input configurations (original features, PCA-transformed, and UMAP-processed) to rigorously evaluate the influence of different feature representations on model performance.

### 2.4. Machine Learning Algorithms

CNN, RF, SVM, LR, and GBT models were run in Python (version 3.6.8), and deep model training was carried out using the Python library Keras.

#### 2.4.1. CNN Model

CNN has emerged as a highly effective and popular tool for feature extraction and model building [29]. Convolutional layers, activation layers, pooling layers, and fully connected layers are all essential components of a CNN [30]. In this experiment, the CNN included an input layer, two convolutional layers (numbers were 64 and 256; kernel sizes were 5 and 3), channel attention and spatial attention mechanisms for feature recalibration, a global max pooling layer, and a multilayer perceptron branch with residual connections. The input layer of the CNN was a one-dimensional vector with 3338 data points. After feature extraction through dual-branch interactions and attention-based refinement, the adulterated CAO was identified through hierarchical projection in fully connected layers with softmax activation.

#### 2.4.2. RF Model

An RF is a collection of numerous trained decision trees, generally based on a bagging algorithm [31]. The RF algorithm presents interesting properties, such as a remarkable capacity for handling mixed or greatly unbalanced datasets, flexibility with no formal assumptions regarding data structure, and the ability to address complex nonlinear systems [32]. In this experiment, features underwent preprocessing, including label encoding, data expansion, normalization, and data splitting (test_size = 0.2, random_state = 42), before the RF algorithm was used. Then, an RF model was constructed (n_estimators = 100, random_state = 42), trained, and used for prediction, and the accuracy of the RF model was calculated.

#### 2.4.3. SVM Model

The SVM model can exhibit good performance with relatively low computational overhead, making it a valuable tool in the machine learning toolkit for spectral data analysis [33]. After undergoing label encoding, data expansion, normalization, and data splitting, an SVM model was constructed (kernel = ‘linear’, class_weight = ‘balanced’, random_state = 42) and trained, and its performance was predicted and evaluated.

#### 2.4.4. LR Model

The LR algorithm is a fundamental supervised learning model used to identify a dependent parameter based on the independent parameters [34]. After label encoding, data expansion, normalization, and data splitting were conducted, an LR model was created, trained, saved, and used for prediction, and its performance was evaluated. Finally, some of the prediction results were output and compared with the test labels.

#### 2.4.5. GBT Model

GBT is a method that is applied in conjunction with another machine learning algorithm, and involves two models (a “weak” learning model, usually a decision tree, and a “strong” learning model, composed of several weak learning models) [35]. GBT is a kind of synthesis algorithm combined with basis function weights, which can form a strong classifier via multiple weak classifiers through the GBT algorithm [36]. After label encoding, data expansion, normalization, and data splitting, a GBT model was constructed (n_estimators = 100, learning_rate = 0.1, max_depth = 3, random_state = 42), trained, and saved. Afterwards, this model was used for prediction, and its accuracy was calculated.

### 2.5. Training and Testing of Machine Learning Models

#### 2.5.1. Qualitative Models

Five classification models of CNN, RF, SVM, LR, and GBT were constructed. The CNN employed 1D convolutional layers to extract local spectral features, along with global pooling and fully connected layers for probability output. The other four models directly processed the reduced-dimensional features. All the models were trained and evaluated on the same training and testing sets to ensure fair comparison.

#### 2.5.2. Quantitative Models

A hybrid neural network model (Conv–Attention–MLP) was used to predict the percentage compositions of five types of oils. The model processed the reduced-dimensional features through two parallel branches: a Multilayer Perceptron (MLP) branch for learning global patterns, and a 1D convolutional branch equipped with attention mechanisms to highlight important local spectral features. Features from both branches were fused and fed into a Softmax output layer. The model was trained using the Adam optimizer with a Mean Squared Error (MSE) loss function. To prevent overfitting, an independent validation set (20% of the training data) was used for hyperparameter tuning and early cessation. The reported accuracies are based on a held-out test set (20% of the total data) that was not used during training or validation.

### 2.6. Classifier and Model Evaluation

#### 2.6.1. Qualitative Model Evaluation

The performance of the classification models was evaluated using a set of standard metrics. For binary classification, four core metrics were calculated, in accordance with the study by Song et al. [7]: accuracy, recall (sensitivity), precision (positive predictive value), and F1-score (the harmonic mean of precision and recall). These were supplemented with the area under the curve (AUC) of the ROC (AUC-ROC) to evaluate model discrimination capability across all decision thresholds. In multiclass classification, class-specific metrics were computed alongside macro-averaged and micro-averaged aggregates. Multiclass AUC values were determined using a one-versus-rest (OvR) approach.

#### 2.6.2. Quantitative Model Evaluation

All samples were used to construct quantitative models for predicting the concentration levels of CAO, COO, OLO, SOO, and SUO. Based on the study of Song et al. [7], quantitative models between the oil concentrations and spectral features were evaluated with the coefficient of determination for calibration ($R_{C}^{2}$), the root mean square error of calibration (RMSEC), the coefficient of determination for prediction $\;(R_{p}^{2})$, the root mean square error of prediction (RMSEP), and the ratio of prediction to deviation (RPD = SD/RMSEP), where SD is calculated from the prediction set. The models were trained using Adam optimization with learning rate decay and early cessation, with performance metrics recorded over 10 independent runs to ensure robustness.

## 3. Results and Discussion

### 3.1. SICRIT-HRMS Fingerprint

**Figure 2:**
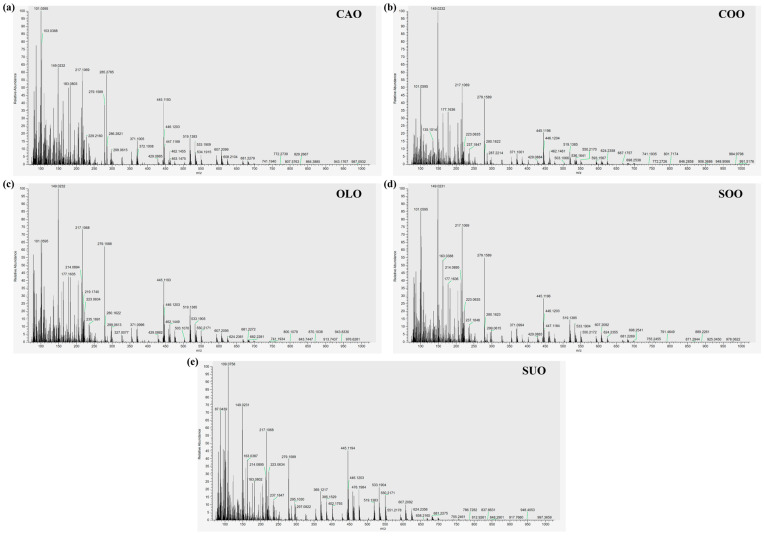
Mass spectra of CAO (**a**), COO (**b**), OLO (**c**), SOO (**d**), and SUO (**e**) generated in positive-ion mode by SICRIT-HRMS measurement. CAO—camellia oil; COO—corn oil; OLO—olive oil; SOO—soybean oil; SUO—sunflower oil.

The representative SICRIT-HRMS fingerprints of the five pure vegetable oils (CAO, COO, OLO, SOO, and SUO) are shown in Figure 2. The volatile and semi-volatile compounds in these oils can largely be found in the relative molecular mass range of 75–700 Da in the mass spectra (Figure 2a–e). Previous studies have shown that the main volatile compounds in CAO are hydrocarbons (10.35%), alcohols (31.71%), aldehydes (44.54%), and esters (3.93%) [37]. The four other vegetable oils (COO, OLO, SOO, and SUO) have also been found to be rich in volatile compounds in varying proportions [38,39]. Although some differences were observed in the mass spectra profiles of these five pure vegetable oils (CAO, COO, OLO, SOO, and SUO), further data mining is still required to improve the classification potential of volatile compounds in them. Notably, the low *m*/*z* region (approximately 100–300) was consistently identified as highly important in subsequent feature selection and regression analyses. This region predominantly corresponds to ions from low-molecular-weight volatile and semi-volatile organic compounds, which are key contributors to the aroma, flavor, and oxidative stability profiles of edible oils [37,39]. The distinct composition of CAO—particularly its higher proportion of alcohols and aldehydes such as nonanal and octanal—compared to common adulterants such as SOO and SUO (which generate different oxidation-derived volatiles, e.g., (E,E)-2,4-decadienal) [38], likely generates differentiable spectral patterns within this *m*/*z* window. Therefore, the feature importance observed in the 100–300 region is underpinned by tangible chemical differences in volatile composition between CAO and potential adulterants, aligning with information on known authenticity markers [3,4,37,38].

Thus, in response to the demand for MS-based discrimination of adulterated CAO, a comprehensive framework for feature extraction and importance analysis was established in this study. To ensure the robustness and relevance of the extracted features, dynamic binning, temporal feature aggregation, chromatographic behavior extraction, and multi-method feature importance evaluation were integrated in this framework. After dynamic binning processing, the binning results of all intervals were concatenated to form the final feature vector. Moreover, to precisely identify adulterated CAO, MS data-based multi-level feature extraction, combined with multiple feature importance analysis techniques, was adopted to ensure the validity and reliability of the features. After feature extraction, the time-aggregated and chromatographic features were fused to form a final feature vector with a total dimension of 3338. In the feature importance analysis stage, a full-range feature acquisition strategy was adopted: the *m*/*z* range from 1 to 1100 was divided into bins with a step size of 0.01, and different importance evaluation methods were utilized for the analysis. For each feature scoring file, only the top five features with the highest scores (corresponding to the *m*/*z* values) were retained, resulting in a total of 240 feature intervals being selected. Subsequently, all the feature points were expanded and sorted, and a small window of ±0.03 around each point was employed as the feature interval. Finally, these intervals were merged to form a final bin configuration with 92 settings.

Similarly, the SICRIT-HRMS fingerprints of the binary, ternary, quaternary, and quinary adulteration systems were acquired. The representative mass spectra (V_B_/V_A_ = 50%:50%, with V_A_ and V_B_ representing the volumes of CAO and adulterated oil, respectively) of these systems generated in positive-ion mode via SICRIT-HRMS measurement are shown in Figures S1–S4 (available in the Supplementary Materials).

### 3.2. Binary Qualitative Modeling for the Identification of Adulterated CAO

**Figure 3:**
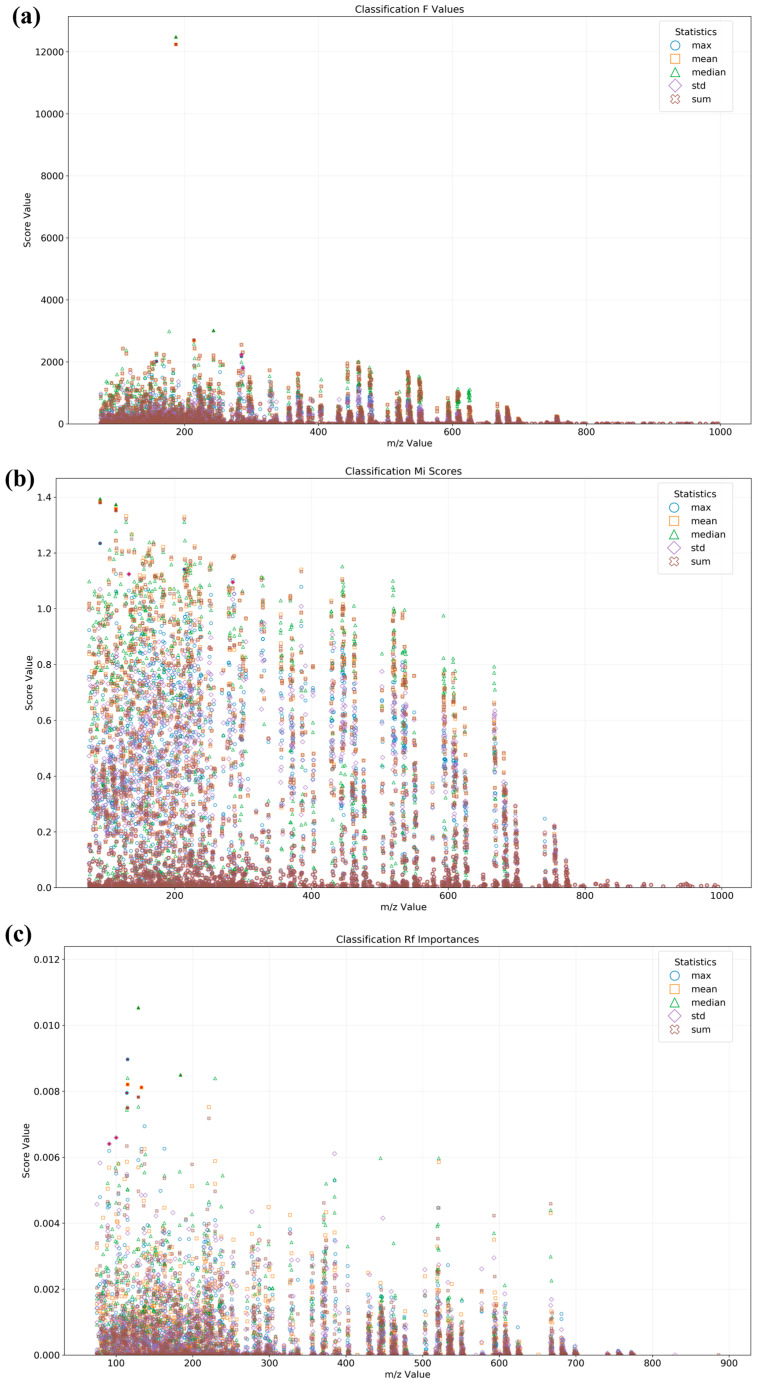
The feature importance score of classification calculated using ANOVA F-value selection (**a**), mutual information ranking (**b**), and Random Forest importance (**c**). Red points indicate selected feature points.

The binary qualitative model is effective in identifying whether CAO is adulterated, and thus suitable for large-scale preliminary identification. Analysis of variance (ANOVA), F-value selection, and mutual information ranking methods have been applied for feature selection in optimizing the classification of pork adulteration in beef [40]. Meanwhile, the Random Forest binary classification method has exhibited considerable superiority in authentication problems relative to partial least squares for discriminant analysis [32]. Thus, ANOVA F-value selection, mutual information ranking, and Random Forest importance methods were used to evaluate the *m*/*z* features for oil classification, as shown in Figure 3. ANOVA F-value selection (Figure 3a) demonstrated that features in the low *m*/*z* region (0~400) exhibited concentrated data distribution with prominent max values, indicating the corresponding features with significant differences among different types of oil might be specific molecular markers. In contrast, the high *m*/*z* region (>600) showed lower statistical significance with minimal classification contribution. Mutual information ranking (Figure 3b) revealed strong feature-class correlations in both the low *m*/*z* (<200) and high *m*/*z* (800~1000) regions, as evidenced by their elevated max values, suggesting these ranges might contain small or large molecular biomarkers, respectively. The high *m*/*z* (800~1000) could be attributed to triglycerides [22]. The intermediate region (*m*/*z* 400~600) displayed moderate mean values, indicating weaker associations. Random Forest importance (Figure 3c) showed that the max and mean values in the *m*/*z* 100~300 range were relatively high, constituting the key classification features, whilst the *m*/*z* > 500 region displayed limited discriminatory power. In general, the low *m*/*z* region (especially 100~300) exhibited importances to oil classification in the above three methods, indicating this region might be the key feature region for identifying oil. The *m*/*z* 256 mass chromatogram was found to mainly contain C2 chrysenes [41]. The typical ions *m*/*z* 190, 203, and 218 have been identified to be δ-amyrin [42]. Mass fragments of *m*/*z* 57, 69, 82, and 83 were identified as straight, branched, or cycloalkanes [43]. Furthermore, the results obtained using the three methods above showed differences, reflecting distinct analytical perspectives and the inherent characteristics of each statistical method.

**Figure 4:**
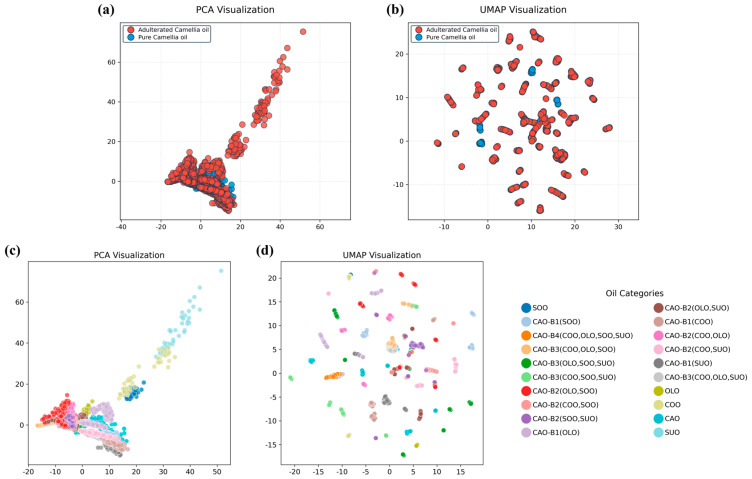
Visualization of PCA and UMAP dimensionality reduction techniques. (**a**,**b**) Binary classification; (**c**,**d**) multiclass classification. CAO—camellia oil; COO—corn oil; OLO—olive oil; SOO—soybean oil; SUO—sunflower oil; PCA—principal component analysis; UMAP—uniform manifold approximation and projection.

Subsequently, the dataset obtained using the binary qualitative model was subjected to dimensionality reduction using PCA and UMAP methods, as shown in Figure 4a,b. PCA reduces the dimensionality of a complex dataset, extracts the most important information according to the spectral features of the tested samples, and identifies outliers in the dataset [44]. In Figure 4a, the adulterated CAO samples (red points) and pure CAO samples (blue points) revealed significant distributional overlap. The pure CAO samples are mainly distributed in regions with low principal component values, whereas the adulterated CAO samples display a stratified distribution along the principal component axes. Obviously, the PCA plot preserved key features while discarding some discriminative information. Thus, it is insufficient for the precise classification of adulterated and pure CAO samples. As PCA does not preserve the local structure, UMAP was developed as an innovative new practical strategy for dimensionality reduction that greatly preserves the local and global structure of the original data [45]. In the UMAP visual plot (Figure 4b), the distributions of the adulterated CAO samples (red points) and pure CAO samples (blue points) show clear cluster structures, and the boundaries for these two types of samples are clear. The pure CAO samples formed relatively independent and discrete clusters, whilst the adulterated CAO samples exhibited a more dispersed distribution. Notably, there are fewer overlapping regions in the UMAP plot than in the PCA plot. Therefore, the UMAP plot was effective in accentuating the feature-space differences between the adulterated and pure CAO samples, thereby allowing for the sample classifications to be intuitively distinguished.

The CNN, RF, SVM, LR, and GBT algorithms have been used to identify oil adulteration [46]. With binary classification, the performances of these five machine learning algorithms in distinguishing pure CAO and adulterated CAO under different dimensionality reduction methods (no dimensionality reduction (NON), PCA, and UMAP) were analyzed. The results are summarized in Table 1. In terms of the NON treatment, the RF, LR, and GBT algorithms achieved perfect classification with respect to the raw data, as they had accuracies of 100.00% and AUC values of 1.0000. These results indicate that the original feature-space already possessed strong discriminative power. In contrast, the SVM algorithm performed poorly with an accuracy of only 86.26%. The CNN algorithm exhibited a minor misclassification with an accuracy of 99.95%; specifically, one pure CAO sample was misclassified as an adulterated CAO sample. Regarding PCA dimensionality reduction, the CNN, RF, and GBT algorithms exhibited accuracies of 100.00%, suggesting that the PCA effectively preserved critical linear discriminative information. Moreover, after feature simplification, the computational efficiencies of these three algorithms improved, whilst their overfitting risks reduced. However, the accuracy of the LR algorithm slightly declined to 98.51%, with 18 false negatives, implying that some nonlinear, subtle features were discarded. The accuracy of the SVM algorithm notably decreased to 81.79%, revealing its sensitivity to nonlinear feature loss. For UMAP dimensionality reduction, the RF and GBT algorithms possessed accuracies of 100.00%, F1-scores of 0.9951, and AUC values of 1.0000, demonstrating their strong adaptability to nonlinear low-dimensional spaces. The performance of the CNN algorithm was a little worse than that of the RF and GBT algorithms, which had 98.70% accuracy and a recall value of 98.21%. This finding can be explained by the convolutional layers’ localized focus on manifold features. In contrast, the accuracies of the LR (81.51%) and SVM (52.21%) algorithms significantly decreased. These two algorithms are greatly dependent on global linear decision boundaries and incompatible with the nonlinear manifolds constructed by UMAP, leading to the failure of the decision boundary.

Overall, the CNN, RF, and GBT algorithms showed high accuracies under NON, PCA, and UMAP dimensionality reductions. In previous studies, the optimal classification accuracy of fluorescence images paired with a CNN model was lower (94.2%) for identifying adulteration of OLO with other vegetable oils [47]. The classification accuracy of the RF algorithm has been proven to be 100% when using three-dimensional fluorescence spectroscopy and machine learning for the rapid detection of adulteration in CAO [48]. In spectral band selection for the nondestructive detection of edible oil adulteration using hyperspectral imaging and chemometric analysis, the GBT algorithm showed 100% training accuracy and 93% validation accuracy [49]. Meanwhile, the LR algorithm exhibited high accuracy under NON treatment and PCA dimensionality reduction. The LR algorithm has been reported to be able to classify pure oil (njangsa seed oil, palm kernel oil, or coconut oil) with an accuracy of 93% using Fourier-transform infrared spectroscopy under PCA dimensionality reduction [50]. In practical applications, if the data exhibit clear linear separability and efficiency and interpretability are prioritized, the combination of no (or PCA) dimensionality reduction with LR or an RF is preferable. Conversely, if the data demonstrate significant nonlinearity and require high accuracy, approaches such as UMAP dimensionality reduction combined with RF, GBT, or CNN are more suitable, and computational resources and training time should also be considered. If the sample size is large, dimensionality reduction (PCA or UMAP) should be prioritized to simplify computations, and then appropriate algorithms should be selected to balance accuracy and efficiency.

### 3.3. Multivariate Qualitative Modeling for the Identification of Adulterated CAO

Moreover, the dataset obtained using the multivariate qualitative model was also subjected to dimensionality reduction using the PCA and UMAP methods, as illustrated in Figure 3c and Figure 4d. As shown in Figure 4c, after PCA dimensionality reduction, the pure oil samples (CAO, COO, OLO, SOO, and SUO) formed independent clusters. The clusters of the binary adulteration systems, including CAO-B1(COO), CAO-B1(OLO), CAO-B1(SOO), and CAO-B1(SUO), were distributed around the pure CAO cluster. The clusters of the ternary adulteration systems, including CAO-B2(COO, OLO), CAO-B2(COO, SOO), CAO-B2(COO, SUO), CAO-B2(OLO, SOO), CAO-B2(OLO, SUO), and CAO-B2(SOO, SUO), were located between the two pure oil clusters. For example, the cluster of CAO-B2(COO, OLO) was located between the cluster of COO and that of OLO. The clusters of the quaternary adulteration systems, including CAO-B3(COO, OLO, SOO), CAO-B3(COO, OLO, SUO), CAO-B3(COO, SOO, SUO), and CAO-B3(OLO, SOO, SUO), were distributed within the triangular area formed by the three pure oil clusters. In contrast, all oil samples in the binary, ternary, quaternary, and quinary adulteration systems showed more dispersed distributions after UMAP dimensionality reduction (Figure 4d).

With multiclass classification, the performances of the five machine learning algorithms (CNN, RF, SVM, LR, and GBT) in distinguishing pure CAO and adulterated CAO under NON, PCA, and UMAP dimensionality reduction methods were analyzed. The results are listed in Table 2. In terms of the NON treatment, the LR algorithm possessed the lowest accuracy (98.44%) among the algorithms, and the RF algorithm had the highest accuracy (99.45%). Regarding PCA dimensionality reduction, the LR algorithm also showed the lowest accuracy (98.35%), while the CNN algorithm exhibited the highest accuracy (99.54%). For UMAP dimensionality reduction, the SVM algorithm had the lowest accuracy (92.50%), whereas the RF algorithm revealed the highest accuracy (96.25%). Overall, the RF algorithm maintained relatively high accuracy while the LR algorithm showed relatively low accuracy under NON, PCA, and UMAP dimensionality reduction methods. The RF algorithm previously demonstrated exceptionally high accuracy (100%) by correctly classifying coconut oil and OLO mixed with COO, SOO, and peanut oil [51].

### 3.4. Data Fusion Combined with Machine Learning Analysis

**Figure 5:**
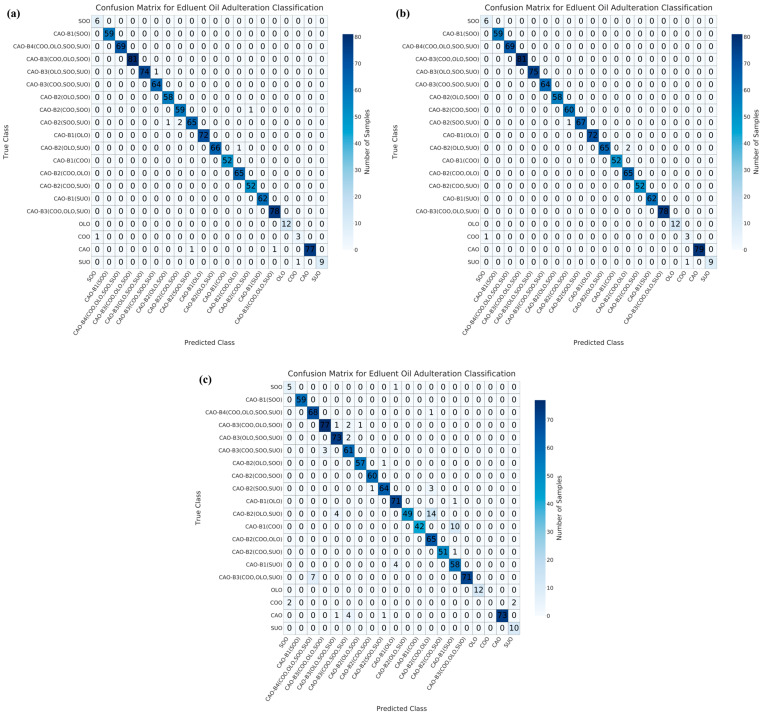
Confusion matrix results for the test set of CNN model with NON (**a**), PCA dimensionality reduction (**b**), and UMAP dimensionality reduction (**c**). CAO—camellia oil; COO—corn oil; OLO—olive oil; SOO—soybean oil; SUO—sunflower oil; PCA—principal component analysis; UMAP—uniform manifold approximation and projection.

**Figure 6:**
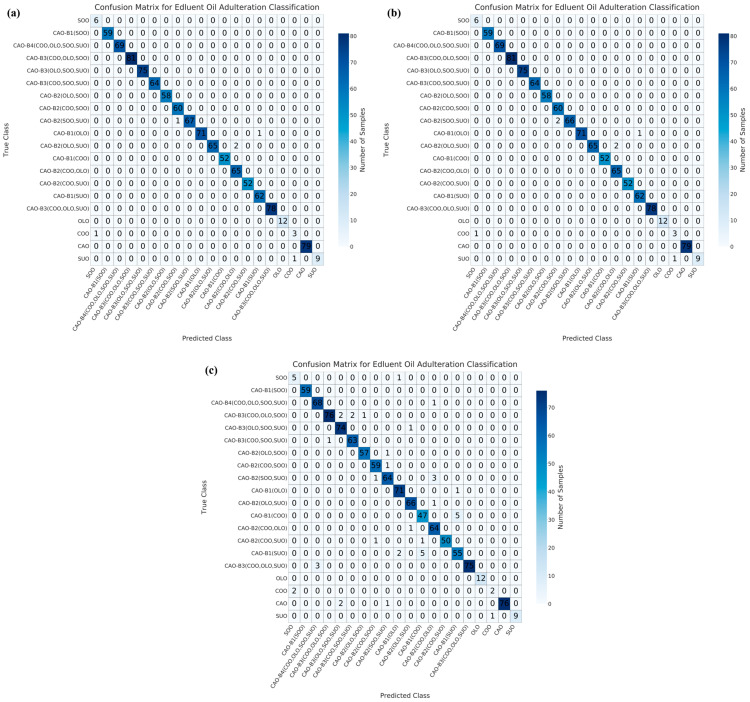
Confusion matrix results for the test set of RF model with NON (**a**), PCA dimensionality reduction (**b**), and UMAP dimensionality reduction (**c**). CAO—camellia oil; COO—corn oil; OLO—olive oil; SOO—soybean oil; SUO—sunflower oil; PCA—principal component analysis; UMAP—uniform manifold approximation and projection.

**Figure 7:**
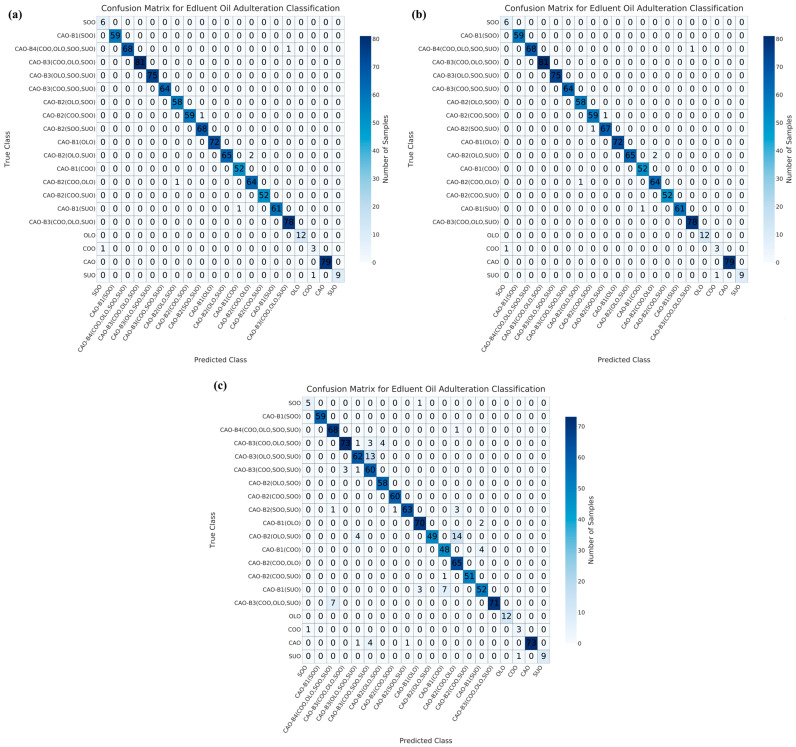
Confusion matrix results for the test set of SVM model with NON (**a**), PCA dimensionality reduction (**b**), and UMAP dimensionality reduction (**c**). CAO—camellia oil; COO—corn oil; OLO—olive oil; SOO—soybean oil; SUO—sunflower oil; PCA—principal component analysis; UMAP—uniform manifold approximation and projection.

**Figure 8:**
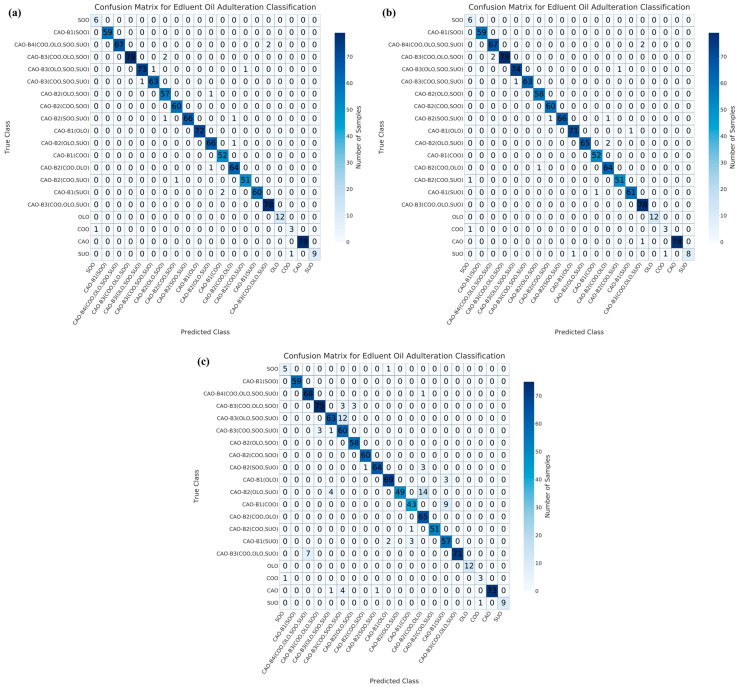
Confusion matrix results for the test set of LR model with NON (**a**), PCA dimensionality reduction (**b**), and UMAP dimensionality reduction (**c**). CAO—camellia oil; COO—corn oil; OLO—olive oil; SOO—soybean oil; SUO—sunflower oil; PCA—principal component analysis; UMAP—uniform manifold approximation and projection.

**Figure 9:**
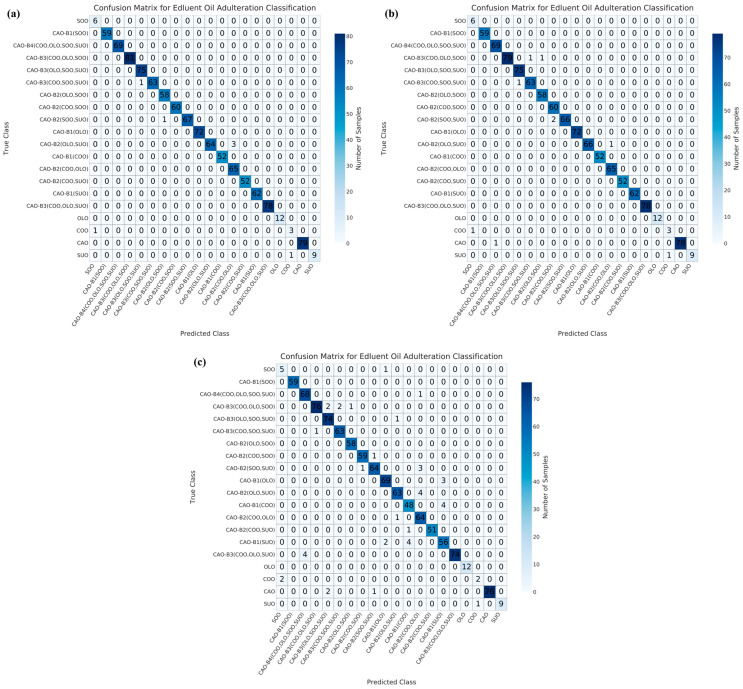
Confusion matrix results for the test set of GBT model with NON (**a**), PCA dimensionality reduction (**b**), and UMAP dimensionality reduction (**c**). CAO—camellia oil; COO—corn oil; OLO—olive oil; SOO—soybean oil; SUO—sunflower oil; PCA—principal component analysis; UMAP—uniform manifold approximation and projection.

Misclassification primarily occurs in multi-adulteration scenarios of CAO within the predefined adulteration settings [52]. To evaluate the performance of the developed models (CNN, RF, SVM, LR, and GBT), 1093 blind samples were used for testing. The confusion matrix results are shown in Figure 5, Figure 6, Figure 7, Figure 8 and Figure 9. Regarding the CNN model, 10, 5, and 67 samples were misclassified under NON (Figure 5a), PCA (Figure 5b), and UMAP (Figure 5c) dimensionality reductions, respectively. Under NON dimensionality reduction (Figure 5a), two pure CAO samples were misclassified as adulterated CAO samples, CAO-B2(SOO, SUO) and CAO-B3(COO, OLO, and SUO), respectively. One pure COO sample and one pure SUO sample were misclassified as SOO and COO samples, respectively. Meanwhile, five samples in ternary adulteration systems (as CAO-B2(SOO, SUO), CAO-B2(COO, SOO), and CAO-B2(OLO, SUO)) and one sample in quaternary adulteration system (as CAO-B3(OLO, SOO, SUO)) were misclassified. Under PCA dimensionality reduction (Figure 5b), one pure COO sample and one pure SUO sample were also misclassified as SOO and COO samples, respectively. Three samples in ternary adulteration systems (as CAO-B2(SOO, SUO) and CAO-B2(OLO, SUO)) were misclassified. Under UMAP dimensionality reduction (Figure 5c), six pure CAO samples were misclassified as adulterated CAO samples: CAO-B2(SOO, SUO), CAO-B3(OLO, SOO, SUO), and CAO-B3(COO, SOO, SUO), respectively. One pure SOO sample was misclassified as a CAO-B1(OLO) sample. Four pure COO samples were misclassified as two SOO and two SUO samples, respectively. Fifteen samples in binary adulteration systems (as CAO-B1(OLO), CAO-B1(COO), and CAO-B1(SUO)), twenty-four ternary samples in adulteration systems (as CAO-B2(OLO, SOO), CAO-B2(SOO, SUO), CAO-B2(OLO, SUO), and CAO-B2(COO, SUO)), sixteen samples in quaternary adulteration systems (as CAO-B3(COO, OLO, SOO), CAO-B3(OLO, SOO, SUO), CAO-B3(COO, SOO, SUO), and CAO-B3(COO, OLO, SOO)), and one sample in quinary adulteration system (as CAO-B4(COO, OLO, SOO, SUO)) were misclassified.

Regarding the RF model, 6, 7, and 41 samples were misclassified under NON (Figure 6a), PCA (Figure 6b), and UMAP (Figure 6c) dimensionality reductions, respectively. Under NON dimensionality reduction (Figure 6a), one pure COO sample and one pure SUO sample were misclassified as SOO and COO samples, respectively. One sample in binary adulteration system (as CAO-B1(OLO)) and three samples in ternary adulteration systems (as CAO-B2(SOO, SUO) and CAO-B2(OLO, SUO)) were misclassified. Under PCA dimensionality reduction (Figure 6b), one pure COO sample and one pure SUO sample were also misclassified as SOO and COO samples, respectively. One sample in binary adulteration system (as CAO-B1(OLO)) and four samples in ternary adulteration systems (as CAO-B2(SOO, SUO) and CAO-B2(OLO, SUO)) were misclassified. Under UMAP dimensionality reduction (Figure 6c), three pure CAO samples were misclassified as adulterated CAO samples, CAO-B2(SOO, SUO) and CAO-B3(OLO, SOO, SUO). One pure SOO sample was misclassified as CAO-B1(OLO). Two COO samples were misclassified as SOO, and one SUO sample was misclassified as COO. Thirteen samples in binary adulteration systems (as CAO-B1(OLO), CAO-B1(COO), and CAO-B1(SUO)), ten samples in ternary adulteration systems (as CAO-B2(COO, OLO), CAO-B2(COO, SOO), CAO-B2(COO, SUO), CAO-B2(OLO, SOO), CAO-B2(OLO, SUO), and CAO-B2(SOO, SUO)), ten samples in quaternary adulteration systems (as CAO-B3(COO, OLO, SOO), CAO-B3(COO, OLO, SUO), CAO-B3(COO, SOO, SUO), and CAO-B3(OLO, SOO, SUO)) and one sample in quinary adulteration system (as CAO-B4(COO, OLO, SOO, SUO)) were misclassified.

For the SVM model, 8, 9, and 82 samples were misclassified under NON (Figure 7a), PCA (Figure 7b), and UMAP (Figure 7c) dimensionality reductions, respectively. Under NON dimensionality reduction (Figure 7a), one pure COO sample and one pure SUO sample were misclassified as SOO and COO samples, respectively. One binary adulteration system (as CAO-B1(SUO)), four samples in ternary adulteration systems (as CAO-B2(COO, SOO), CAO-B2(OLO, SUO), and CAO-B2(COO, OLO)), and one sample in quinary adulteration system (as CAO-B4(COO, OLO, SOO, SUO)) were misclassified. Under PCA dimensionality reduction (Figure 7b), one pure COO sample and one pure SUO sample were also misclassified as SOO and COO samples, respectively. One sample in binary adulteration system (as CAO-B1(SUO)), five samples in ternary adulteration systems (as CAO-B2(COO, SOO), CAO-B2(SOO, SUO), CAO-B2(OLO, SUO), and CAO-B2(COO, OLO)) and one sample in quinary adulteration system (as CAO-B4(COO, OLO, SOO, SUO)) were misclassified. Under UMAP dimensionality reduction (Figure 7c), six pure CAO samples were misclassified as adulterated CAO samples, CAO-B3(OLO, SOO, SUO), CAO-B3(COO, SOO, SUO), and CAO-B2(SOO, SUO). One pure SOO sample was misclassified as CAO-B1(OLO). One pure COO sample and one pure SUO sample were also misclassified as SOO and COO samples, respectively. Sixteen samples in binary adulteration systems (as CAO-B1(OLO), CAO-B1(COO), and CAO-B1(SUO)), twenty-four samples in ternary adulteration systems (as CAO-B2(SOO, SUO), CAO-B2(OLO, SUO), and CAO-B2(COO, SUO)), thirty-two samples in quaternary adulteration systems (as CAO-B3(COO, OLO, SOO), CAO-B3(COO, OLO, SUO), CAO-B3(COO, SOO, SUO), and CAO-B3(OLO, SOO, SUO)) and one sample in quinary adulteration system (as CAO-B4(COO, OLO, SOO, SUO)) were misclassified.

Regarding the LR model, 17, 18, and 78 samples were misclassified under NON (Figure 8a), PCA (Figure 8b), and UMAP (Figure 8c) dimensionality reductions, respectively. Under NON dimensionality reduction (Figure 8a), one pure COO sample and one pure SUO sample were misclassified as SOO and COO samples, respectively. Two samples in binary adulteration systems (as CAO-B1(SUO)), six samples in ternary adulteration systems (as CAO-B2(OLO, SOO), CAO-B2(SOO, SUO), CAO-B2(OLO, SUO), CAO-B2(COO, OLO), and CAO-B2(COO, SUO)), five samples in quaternary adulteration systems (as CAO-B3(COO, OLO, SOO), CAO-B3(OLO, SOO, SUO), and CAO-B3(COO, SOO, SUO)) and two samples in quinary adulteration systems (as CAO-B4(COO, OLO, SOO, SUO)) were misclassified. Under PCA dimensionality reduction (Figure 8b), one pure CAO sample was misclassified as an adulterated CAO sample (CAO-B3(COO, OLO, SUO)), and one SUO sample was misclassified as CAO-B1(OLO). One pure COO sample and one pure SUO sample were also misclassified as SOO and COO samples, respectively. Two samples in binary adulteration systems (as CAO-B1(OLO) and CAO-B1(SUO)), six samples in ternary adulteration systems (as CAO-B2(SOO, SUO), CAO-B2(OLO, SUO), CAO-B2(COO, OLO), and CAO-B2(COO, SUO)), four samples in quaternary adulteration systems (as CAO-B3(COO, OLO, SOO), CAO-B3(OLO, SOO, SUO), and CAO-B3(COO, SOO, SUO) and two samples in quinary adulteration systems (as CAO-B4(COO, OLO, SOO, SUO)) were misclassified. Under UMAP dimensionality reduction (Figure 8c), six pure CAO samples were misclassified as adulterated CAO samples (CAO-B3(OLO, SOO, SUO), CAO-B3(COO, SOO, SUO), and CAO-B2(SOO, SUO)), and one pure SOO sample was misclassified as CAO-B1(OLO). One pure COO sample and one pure SUO sample were also misclassified as SOO and COO samples, respectively. Seventeen samples in binary adulteration systems (as CAO-B1(OLO), CAO-B1(COO), and CAO-B1(SUO)), twenty-three samples in ternary adulteration systems (as CAO-B2(SOO, SUO), CAO-B2(OLO, SUO), and CAO-B2(COO, SUO)), twenty-nine samples in quaternary adulteration systems (as CAO-B3(COO, OLO, SOO), CAO-B3(COO, OLO, SUO), CAO-B3(COO, SOO, SUO), and CAO-B3(OLO, SOO, SUO)) and one sample in quinary adulteration system (as CAO-B4(COO, OLO, SOO, SUO)) were misclassified.

In terms of the GBT model, 7, 9, and 43 samples were misclassified under NON (Figure 9a), PCA (Figure 9b), and UMAP (Figure 9c) dimensionality reductions, respectively. Under NON dimensionality reduction (Figure 9a), one pure COO sample and one pure SUO sample were misclassified as SOO and COO samples, respectively. Four samples in ternary adulteration systems (as CAO-B2(SOO, SUO) and CAO-B2(OLO, SUO)) and one sample in quaternary adulteration system (as CAO-B3(COO, SOO, SUO)) were misclassified. Under PCA dimensionality reduction (Figure 9b), one pure CAO sample was misclassified as CAO-B4(COO, OLO, SOO, SUO). One pure COO sample and one pure SUO sample were also misclassified as SOO and COO samples, respectively. Three samples in ternary adulteration systems (as CAO-B2(SOO, SUO) and CAO-B2(OLO, SUO)) and three samples in quaternary adulteration systems (as CAO-B3(COO, OLO, SOO) and CAO-B3(COO, SOO, SUO)) were misclassified. Under UMAP dimensionality reduction (Figure 9c), three pure CAO samples were misclassified as adulterated CAO samples (CAO-B3(OLO, SOO, SUO) and CAO-B2(SOO, SUO)), and one pure SOO sample was misclassified as adulterated CAO-B1(OLO). Two pure COO samples and one pure SUO sample were misclassified as SOO and COO samples, respectively. Thirteen samples in binary adulteration systems (as CAO-B1(OLO), CAO-B1(COO), and CAO-B1(SUO)), eleven samples in ternary adulteration systems (as CAO-B2(COO, SOO), CAO-B2(SOO, SUO), CAO-B2(OLO, SUO), CAO-B2(COO, OLO), and CAO-B2(COO, SUO)), eleven samples in quaternary adulteration systems (as CAO-B3(COO, OLO, SOO), CAO-B3(COO, OLO, SUO), CAO-B3(COO, SOO, SUO), and CAO-B3(OLO, SOO, SUO)) and one sample in quinary adulteration system (as CAO-B4(COO, OLO, SOO, SUO)) were misclassified.

Overall, the RF model showed the lowest misclassifications (54 samples), followed by the GBT model (59 samples), under NON, PCA, and/or UMAP dimensionality reductions. Regarding the binary adulteration system, the CNN and GBT models showed the best performances under NON or PCA dimensionality reductions, while the RF and GBT models exhibited the best performances under UMAP dimensionality reduction. For the ternary adulteration system, the RF, CNN/GBT (followed by RF), and RF models presented the best performances under NON, PCA, and UMAP dimensionality reductions, respectively. For the quaternary adulteration system, the RF and SVM models exhibited the best performances under NON dimensionality reduction; the CNN, RF, and SVM models revealed the best performances under PCA dimensionality reduction; and the RF model showed the best performance under UMAP dimensionality reduction. For the quinary adulteration system, the CNN, RF, and GBT models expressed the best performances under NON and PCA dimensionality reductions, whereas the CNN, RF, SVM, LR, and GBT models exhibited the same performance under UMAP dimensionality reduction. Therefore, the GBT model under NON, PCA, and/or UMAP dimensionality reductions is preferable for a binary adulteration system. Previously, the GBT model was proven to be capable of achieving a 100% recognition rate for identifying adulterated safflower seed oil via hyperspectral spectroscopy [53]. The RF model under NON, PCA, and/or UMAP dimensionality reductions can be tentatively recommended for ternary and quaternary adulteration systems. In a previous study, the RF model was found to outperform others in the qualitative detection of CAO adulteration using an electronic nose based on wavelet decomposition humidity correction [54]. The CNN, RF, and GBT models were effective for the quinary adulteration system under NON and PCA dimensionality reductions.

### 3.5. Quantitative Modeling for Adulteration Level Prediction of Adulterated CAO

**Figure 10:**
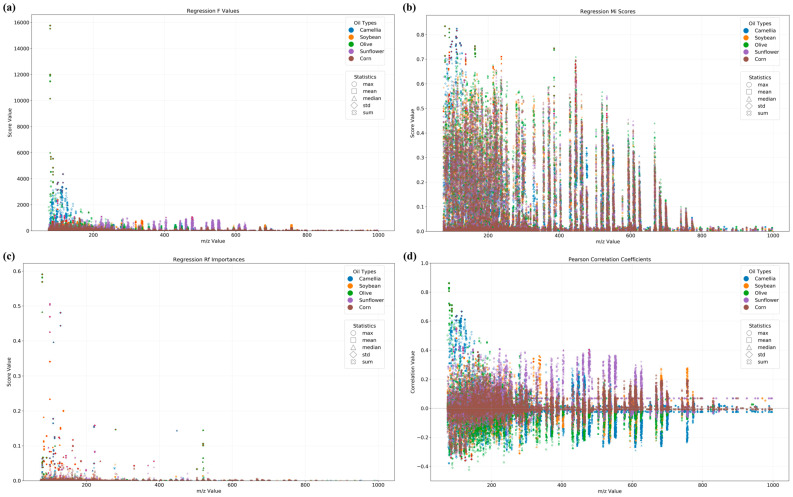
The feature importance score of regression (ANOVA F-value selection (**a**), mutual information (MI) ranking (**b**), and Random Forest importance (**c**)) and the Pearson correlation coefficients (**d**). Red points indicate selected feature points.

To realize oil quantitative classification, ANOVA F-value selection (Figure 10a), mutual information ranking (Figure 10b), and Random Forest importance (Figure 10c) methods were used to evaluate the feature importance score of regression. Meanwhile, a Pearson’s correlation coefficient analysis (Figure 10d) was performed. As shown in Figure 10a, both CAO (blue) and SOO (orange) demonstrated a pronounced accumulation of high F-value signals within the *m*/*z* 200–400 range, indicating this region was a definitive natural differentiation core zone for these two oils. As illustrated in Figure 10b, all the oils exhibited notably higher M-values within the *m*/*z* 200–600 range, implying this region was a crucial information association core zone where spectral features had optimal discriminative capacity for oil classification. As shown in Figure 10c, both CAO (blue) and SOO (orange) showed obviously higher R-values within the *m*/*z* 200–400 range, suggesting this region was particularly critical for authentication and adulteration detections for these two oils. As displayed in Figure 10d, CAO (blue) exhibited consistently positive correlation coefficients (absolute values > 0.4) within the *m*/*z* 200–400 range, hinting at a linear relationship between signal intensity in this region and CAO content percentage. This quantitative correlation suggests that the spectral features could serve as reliable indicators for CAO quantification in potential adulteration scenarios.

CNN models have been widely used to quantitatively predict adulteration levels in camellia-blended oil [48,55,56]. In order to evaluate the generalization ability of the CNN quantitative model, 80% of the samples were randomly selected as the training set, with the remaining 20% serving as the prediction set. Within the training set, 80% and 20% were used for training and validation, respectively. The programs were independently executed in 10 runs under three conditions: NON, PCA, and UMAP dimensionality reductions. The relevant statistical results regarding the CNN model are summarized in Table 3. As shown, the PCA-CNN model performed optimally in the quantitative analysis of adulteration levels of CAO.

The quantitative performance of the models was evaluated using several standard metrics, including $R_{C}^{2}$, $R_{P}^{2}$, RMSEC, RMSEP, and RPD. *R*^2^ ($R_{C}^{2}$ and $R_{P}^{2}$) values close to 1 indicate that the model explains most of the variance in the data, with high a $R_{P}^{2}$ signifying strong generalizability to unseen samples. RMSEC and RMSEP quantify the average prediction error, with lower values denoting higher precision; notably, RMSEP is a more stringent measure of practical utility as it is calculated on an independent test set. According to widely accepted guidelines in chemometrics [57,58], an RPD value greater than 3.0 indicates a model has excellent predictive ability suitable for practical applications. In this study, all RPD values reported in Table 3 and Table 4 substantially exceed this threshold (ranging from 5.0 to 16.3). This finding confirms that our PCA-CNN model, especially in terms of predicting OLO and SUO adulteration levels, meets the criteria for a robust quantitative predictive model. The high $R_{P}^{2}$ values (0.9599–0.9963) and low RMSEP values (1.1–5.8%) further support the model’s accuracy and precision in quantifying adulteration levels. These results demonstrate that the developed approach is not only statistically significant but also practically applicable for quality control purposes.

Based on the results in Table 3, the PCA-CNN model was further employed for the quantitative detection of adulterants (COO, OLO, SOO, and SUO) in CAO. The statistical results are shown in Table 4. Compared to the other models, the PCA-CNN model showed optimal performance in quantitatively detecting OLO in CAO, indicating the prediction accuracy of the developed PCA-CNN model for the adulteration ratio of OLO is extremely high. This superior performance can be attributed to the high compatibility between OLO’s distinctive volatile composition and the feature extraction mechanism of PCA. OLO contains high concentrations of characteristic compounds, such as C6 and C5 derivatives from the lipoxygenase pathway, which are closely associated with its sensory and chemical profiles [59]. These compounds produce strong, concentration-linear signals in the low *m*/*z* range (100–400 Da) in mass spectrometry (Figure 10d)—a linear trend effectively captured by PCA. Consequently, the principal components extracted by PCA can accurately reflect the compositional variations between OLO and CAO with minimal feature overlap, thereby enhancing the predictive performance of the PCA-CNN framework. Furthermore, the PCA-CNN model exhibited the second-highest precision for the quantitative detection of SUO in CAO. Similarly, the CNN model has been demonstrated to enhance the accuracy of OLO adulteration detection from spectral data [60]. In the study by Liu et al. [61], the SVM model performed best for predicting adulteration of CAO with SOO, and the RF model was optimal for CAO adulterated with COO, rapeseed oil, or peanut oil when utilizing UV-Vis-NIR spectroscopy combined with feature selection methods.

Regarding the practical detection limits of the proposed method, the lowest adulteration level prepared and tested in this study was 3% (*v*/*v*). As demonstrated by the high binary classification accuracies (Table 1) and the low RMSEP values achieved in quantitative prediction (Table 4), the method showed reliable detection and quantification abilities at this level.

## 4. Conclusions

Developing novel techniques for detecting CAO adulteration is of great significance in maintaining the orderly development of the CAO industry and market health. In the present study, a rapid and precise method for identifying and quantifying multi-species vegetable oil adulteration in CAO was established using SICRIT-HRMS and machine learning methods. SICRIT-HRMS fingerprints of CAO, along with its adulterated oils (binary, ternary, quaternary, and quinary adulteration systems), were successfully acquired. In the mass spectra, the low *m*/*z* region (especially 100–300) exhibited importances to oil classification in ANOVA F-value selection, mutual information ranking, and Random Forest importance methods. For qualitative detection, binary classification models based on the CNN, RF, and GBT algorithms showed high accuracies for identifying CAO adulteration under NON, PCA, and UMAP dimensionality reductions. UMAP dimensionality reduction was effective in accentuating the feature-space differences between adulterated and pure CAO samples. In multiclass classification, the RF algorithm exhibited relatively high accuracy in distinguishing pure and adulterated CAO under NON, PCA, and UMAP dimensionality reduction methods. The five developed models (CNN, RF, SVM, LR, and GBT) exhibited different performances: (i) the GBT model under NON, PCA, and/or UMAP dimensionality reductions is preferable for binary adulteration system; (ii) the RF model under NON, PCA, and/or UMAP dimensionality reductions can largely be recommended for ternary and quaternary adulteration system; (iii) the CNN, RF, and GBT models are effective for quinary adulteration system under NON and PCA dimensionality reductions. For quantitative prediction, the PCA-CNN model performed optimally in the quantitative analysis of adulteration levels of CAO. In particular, the PCA-CNN model exhibited optimal performance in the quantitative detection of OLO in CAO, and exhibited the second-highest precision in the quantitative detection of SUO in CAO. In summary, this study presents a non-targeted, efficient, and scalable framework for CAO authentication with multi-species vegetable oils. The findings offer a promising tool for real-world screening and quality control in the edible oil industry. While the models performed well on our current dataset, their broader applicability must be further validated. It should be noted that this method primarily relies on volatile and semi-volatile compound profiles. For oils that have undergone deep refining processes—which may significantly diminish characteristic volatile markers—or those possessing volatile fingerprints highly similar to those of CAO, the detection sensitivity of this approach could be compromised, leading to a potential risk of false negatives. In future work, we will explicitly incorporate oils corresponding to a wider range of geographical origins, cultivars, and processing conditions to rigorously test and enhance the global applicability and robustness of the proposed framework. Furthermore, recognizing that oil adulteration is a complex, multi-component challenge, we plan to extend our analysis to non-volatile constituents and integrate complementary analytical approaches. This integrated strategy is expected to enable a more comprehensive, multifaceted assessment, ultimately strengthening the reliability and applicability of the method for real-world quality control and regulatory screening.

## Figures and Tables

**Table 1:** Results of binary classification models.

Dimensionality Reduction Method	Model	Accuracy/%	Precision/%	Recall/%	F1-Score	AUC
NON	CNN	99.95	99.91	100.00	0.9996	1.0000
	RF	100.00	100.00	100.00	1.0000	1.0000
	SVM	86.26	83.35	91.94	0.8743	0.9449
	LR	100.00	100.00	100.00	1.0000	1.0000
	GBT	100.00	100.00	100.00	1.0000	1.0000
PCA	CNN	100.00	100.00	100.00	1.0000	1.0000
	RF	100.00	100.00	100.00	1.0000	1.0000
	SVM	81.79	87.49	75.81	0.8123	0.8967
	LR	98.51	98.74	98.39	0.9856	0.9997
	GBT	100.00	100.00	100.00	1.0000	1.0000
UMAP	CNN	98.70	99.28	98.21	0.9874	0.9958
	RF	99.49	99.20	99.82	0.9951	0.9952
	SVM	52.21	53.68	58.78	0.5612	0.5890
	LR	81.51	86.80	75.99	0.8103	0.8657
	GBT	99.49	99.20	99.82	0.9951	0.9965

Note: NON—no dimensionality reduction; PCA—principal component analysis; UMAP—uniform manifold approximation and projection; CNN—convolutional neural networks; RF—Random Forest; SVM—support vector machines; LR—logistic regression; GBT—gradient boosting trees; AUC—area under the curve.

**Table 2:** Results of multiclass classification models.

Dimensionality Reduction Method	Model	Accuracy/%	Precision/%	Recall/%	F1-Score	AUC
NON	CNN	99.09	97.40	97.68	0.9749	1.0000
	RF	99.45	99.47	99.45	0.9945	1.0000
	SVM	99.27	99.29	99.27	0.9927	1.0000
	LR	98.44	98.48	98.44	0.9845	0.9999
	GBT	99.36	99.39	99.36	0.9936	1.0000
PCA	CNN	99.54	97.80	98.03	0.9787	0.9998
	RF	99.36	99.39	99.36	0.9936	1.0000
	SVM	99.18	99.20	99.18	0.9918	1.0000
	LR	98.35	98.42	98.35	0.9836	0.9999
	GBT	99.18	99.20	99.18	0.9918	1.0000
UMAP	CNN	93.87	88.53	89.48	0.8867	0.9965
	RF	96.25	96.30	96.25	0.9624	0.9938
	SVM	92.50	93.26	92.50	0.9253	0.9957
	LR	92.77	93.54	92.77	0.9281	0.9955
	GBT	96.07	96.17	96.07	0.9607	0.9967

Note: NON—no dimensionality reduction; PCA—principal component analysis; UMAP—uniform manifold approximation and projection; CNN—convolutional neural networks; RF—Random Forest; SVM—support vector machines; LR—logistic regression; GBT—gradient boosting trees; AUC—area under the curve.

**Table 3:** Statistics results of machine learning modeling of adulteration levels of CAO (mean ± SD).

Model	Training Datasets		Prediction Datasets		RPD
	$R_{C}^{2}$	RMSEC (%)	$R_{P}^{2}$	RMSEP (%)	
NON-CNN	0.9948 ± 0.0012	2.1 ± 0.2	0.9867 ± 0.0012	3.3 ± 0.2	8.7 ± 0.4
PCA-CNN	0.9958 ± 0.0004	1.9 ± 0.1	0.9937 ± 0.0012	2.3 ± 0.2	12.7 ± 1.1
UMAP-CNN	0.9664 ± 0.0017	5.3 ± 0.1	0.9599 ± 0.0022	5.8 ± 0.2	5.0 ± 0.1

Note: NON—no dimensionality reduction; PCA—principal component analysis; UMAP—uniform manifold approximation and projection; CNN—convolutional neural networks. $R_{C}^{2}$—coefficient of determination for calibration; RMSEC—root mean square error of calibration; $R_{p}^{2}$—coefficient of determination for prediction; RMSEP—root mean square error of prediction; RPD = SD/RMSEP.

**Table 4:** Statistical analysis results of PCA-CNN modeling for quantitative detection of adulterants in CAO (mean ± SD).

Types	Training Datasets		Prediction Datasets		RPD
	$R_{C}^{2}$	RMSEC (%)	$R_{P}^{2}$	RMSEP (%)	
COO	0.9845 ± 0.0021	2.2 ± 0.2	0.9765 ± 0.0061	2.6 ± 0.3	6.7 ± 0.8
OLO	0.9974 ± 0.0001	0.9 ± 0.0	0.9963 ± 0.0002	1.1 ± 0.0	16.3 ± 0.4
SOO	0.9844 ± 0.0022	2.2 ± 0.2	0.9794 ± 0.0056	2.4 ± 0.3	7.1 ± 0.9
SUO	0.9965 ± 0.0002	1.0 ± 0.0	0.9901 ± 0.0062	1.7 ± 0.5	11.3 ± 3.1

Note: CAO—camellia oil; COO—corn oil; OLO—olive oil; SOO—soybean oil; SUO—sunflower oil; $R_{C}^{2}$—coefficient of determination for calibration; RMSEC—root mean square error of calibration; $R_{p}^{2}$—coefficient of determination for prediction; RMSEP—root mean square error of prediction; RPD = SD/RMSEP.

## Data Availability

The original contributions presented in the study are included in the article/Supplementary Material; further inquiries can be directed to the corresponding authors.

## Supplementary Materials

The following supporting information can be downloaded at: https://www.mdpi.com/article/10.3390/foods15030434/s1, Table S1: Sample information of the selected vegetable oils. Table S2: Details of all adulterated CAO samples with specific adulteration ratios (V/V, %). Figure S1: Representative mass spectra (V_B_/V_A_ = 50%:50%) of binary adulteration system generated in positive ion mode by SICRIT-HRMS measurement. Figure S2: Representative mass spectra (V_B_/V_A_ = 50%:50%) of ternary adulteration system generated in positive ion mode by SICRIT-HRMS measurement. Figure S3: Representative mass spectra (V_B_/V_A_ = 50%:50%) of quaternary adulteration system generated in positive ion mode by SICRIT-HRMS measurement. Figure S4: Representative mass spectra (V_B_/V_A_ = 50%:50%) of quinary adulteration system generated in positive ion mode by SICRIT-HRMS measurement.
