# Supplementary material for: Adulteration Detection of Multi-Species Vegetable Oils in Camellia Oil Using SICRIT-HRMS and Machine Learning Methods
Source: Foods. 2026 Jan 24;15(3):434. doi: 10.3390/foods15030434 (PMC12896747; doi:10.3390/foods15030434)
Supplement: Supplementary file 1 [file foods-15-00434-s001.zip › foods-4075366-supplementary.pdf]

Table S1 Sample information of the selected vegetable oils

Oil type	Brand	Place of origin	Number
**CAO**	Obtained by hydraulic cold pressing in our laboratory	Jiangxi, China	77
	Local workshop	Guangxi, China	14
	Local workshop	Hunan, China	8
	Tianyu	Jiangxi, China	2
	Runxin	Jiangxi, China	2
	Ganmulin	Jiangxi, China	2
	Qiyunshan	Jiangxi, China	1
	Zidefu	Jiangxi, China	1
**COO**	Jinlongyu	Guangdong, China	2
	Xiwang	Shandong, China	2
	Fulinmen	Jiangsu, China	1
	Fulinmen	Anhui, China	1
	Sanjiu	Heilongjiang, China	1
	Kuiwang	Jiangsu, China	1
	Changkang	Hunan, China	1
	Youcai	Jiangsu, China	1
	Daomai	Guangdong, China	1
	Luhua	Hubei, China	1
	Duoli	Fujian, China	1
	Changshouhua	Shandong, China	1
	Jinzhangmen	Tianjin, China	1
**OLO**	Calena	Fujian, China	2
	Grandpa's Farm	Guangdong, China	1
	Mueloliva	Shanghai, China	1
	Borges	Beijing, China	1
	Aortilee	Jiangxi, China	1
	Aolilanke	Jiangxi, China	1
	Betis	Andalusia, Spain	1
	Bainiankunlun	Gansu, China	1
	Oleo Bella	Neimenggu, China	1
	Agric	Jiangsu, China	1
	Agric	Beijing, China	1
	Olivoila	Shanghai, China	1
	Ouweili	Guangdong, China	1
	Azeite Royal	Shandong, China	1
**SOO**	Jiusan	Heilongjiang, China	4
	Jinlongyu	Hebei, China	3
	Beidahuang	Jiangsu, China	1
	Fulinmen	Jiangsu, China	1
	Longda	Shandong, China	1
	Luhua	Jiangsu, China	1
	Dongbeiyikenongxin	Jilin, China	1
	Fengyuan	Shandong, China	1
	Luhua	Liaoning, China	1
	Shengfudi	Shandong, China	1
**SUO**	Kuiwang	Jiangsu, China	2
	Riqing	Jiangsu, China	1

	Zhonglingchucui	Jiangsu, China	1
	Jinlu	Neimenggu, China	1
	Jiusan	Heilongjiang, China	1
	Luhua	Hubei, China	1
	Duoli	Fujian, China	1
	Fulinmen	Anhui, China	1
	Duoli	Jiangsu, China	1
	Fulinmen	Jiangsu, China	1
	Jinlongyu	Hebei, China	1
	Youcai	Jiangsu, China	1
	Jinsheng	Shandong, China	1
	Yuhuang	Shandong, China	1

Note: CAO, camellia oil; COO, corn oil; OLO, olive oil; SOO, soybean oil; SUO, sunflower oil.

Table S2 Details of all adulterated CAO samples with specific adulteration ratios (V/V, %)

CAO (A) adulterated with other oil (B)	Adulteration ratio (V_B/V_A, %)										
	3: 97	5: 95	10: 90	20: 80	30: 70	40: 60	50: 50	60: 40	70: 30	80: 20	90: 10
CAO-B1(COO)	n=3	n=3	n=3	n=3	n=3	n=3	n=3	n=3	n=3	n=3	n=3
CAO-B1(OLO)	n=3	n=3	n=3	n=3	n=3	n=3	n=3	n=3	n=3	n=3	n=3
CAO-B1(SOO)	n=3	n=3	n=3	n=3	n=3	n=3	n=3	n=3	n=3	n=3	n=3
CAO-B1(SUO)	n=3	n=3	n=3	n=3	n=3	n=3	n=3	n=3	n=3	n=3	n=3
CAO-B2(COO, OLO)	n=3	n=3	n=3	n=3	n=3	n=3	n=3	n=3	n=3	n=3	n=3
CAO-B2(COO, SOO)	n=3	n=3	n=3	n=3	n=3	n=3	n=3	n=3	n=3	n=3	n=3
CAO-B2(COO, SUO)	n=3	n=3	n=3	n=3	n=3	n=3	n=3	n=3	n=3	n=3	n=3
CAO-B2(OLO, SOO)	n=3	n=3	n=3	n=3	n=3	n=3	n=3	n=3	n=3	n=3	n=3
CAO-B2(OLO, SUO)	n=3	n=3	n=3	n=3	n=3	n=3	n=3	n=3	n=3	n=3	n=3
CAO-B2(SOO, SUO)	n=3	n=3	n=3	n=3	n=3	n=3	n=3	n=3	n=3	n=3	n=3
CAO-B3(COO, OLO, SOO)	n=3	n=3	n=3	n=3	n=3	n=3	n=3	n=3	n=3	n=3	n=3
CAO-B3(COO, OLO, SUO)	n=3	n=3	n=3	n=3	n=3	n=3	n=3	n=3	n=3	n=3	n=3
CAO-B3(COO, SOO, SUO)	n=3	n=3	n=3	n=3	n=3	n=3	n=3	n=3	n=3	n=3	n=3
CAO-B3(OLO, SOO, SUO)	n=3	n=3	n=3	n=3	n=3	n=3	n=3	n=3	n=3	n=3	n=3
CAO-B4(COO, OLO, SOO, SUO)	n=3	n=3	n=3	n=3	n=3	n=3	n=3	n=3	n=3	n=3	n=3

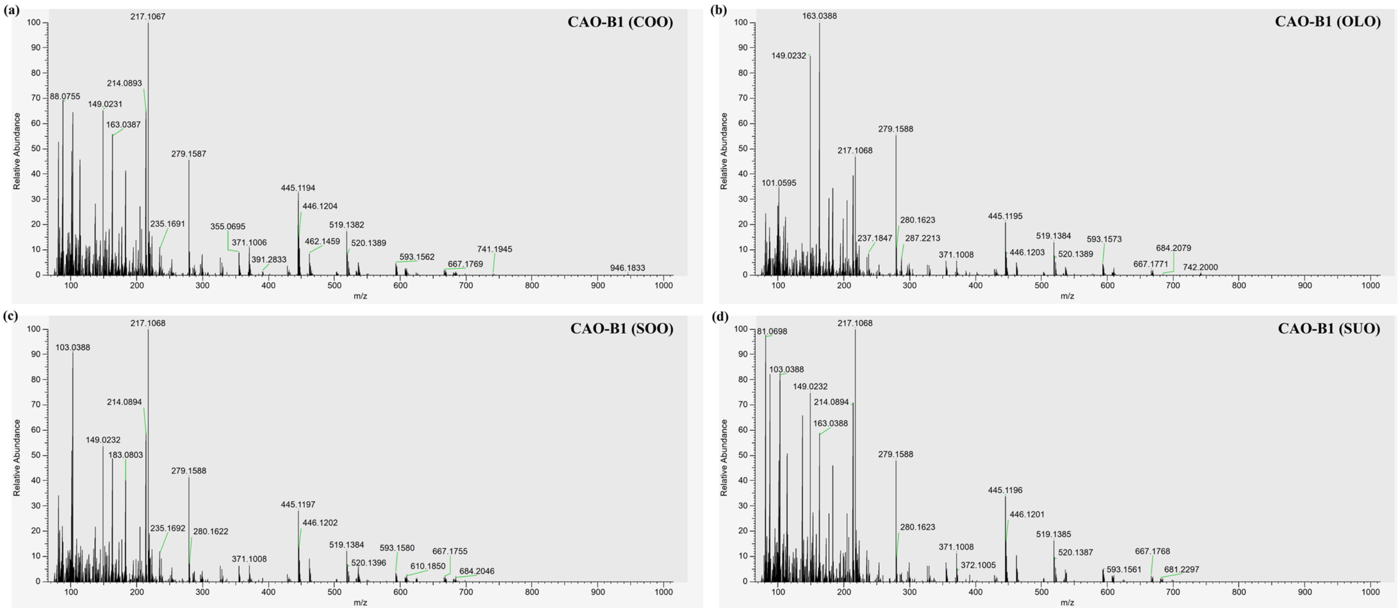

Figure S1 Representative mass spectra (V_B/V_A=50%: 50%) of binary adulteration system generated in positive ion mode by SICRIT-HRMS measurement.

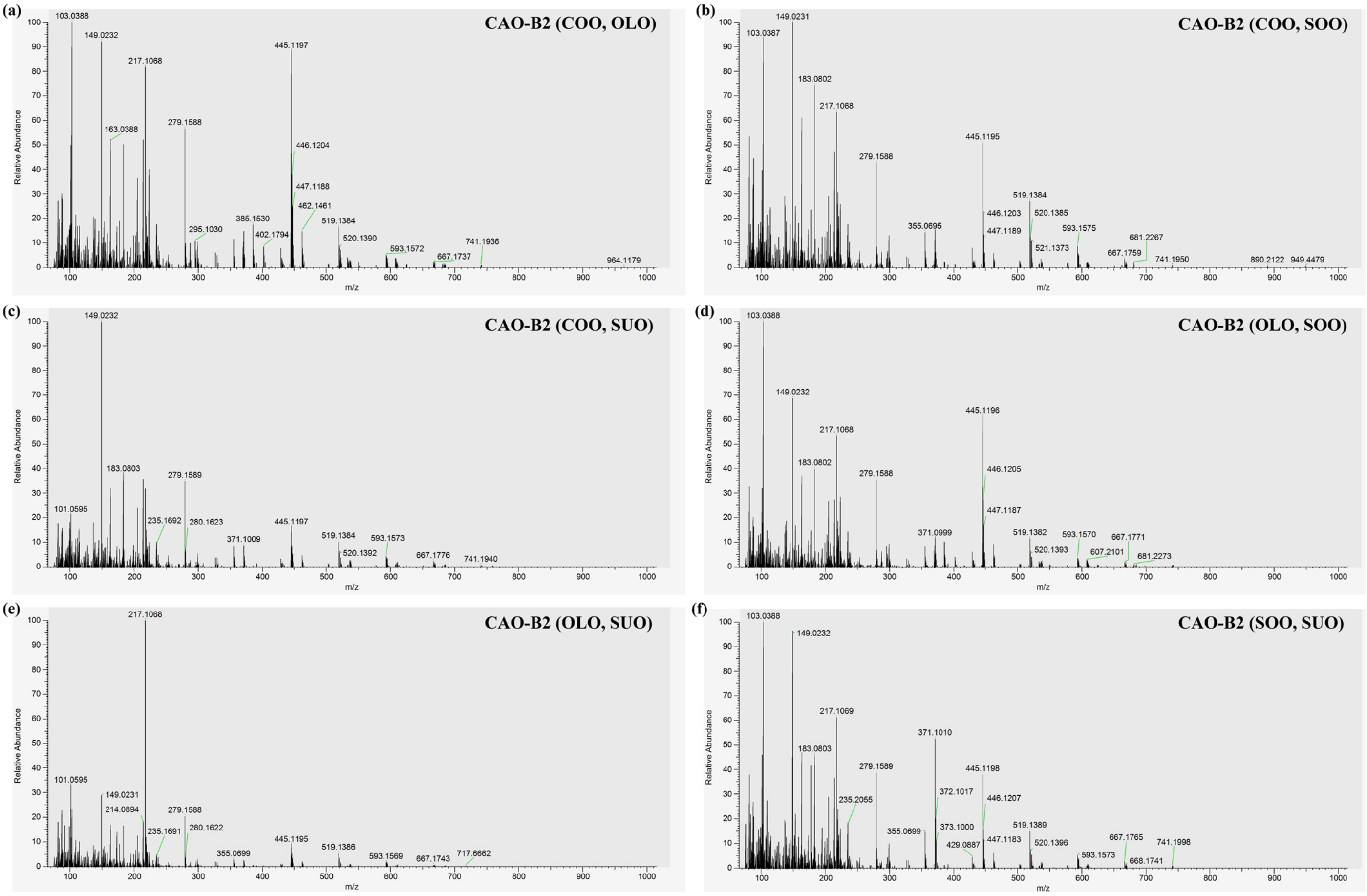

Figure S2 Representative mass spectra (V_B/V_A=50%: 50%) of ternary adulteration system generated in positive ion mode by SICRIT-HRMS measurement.

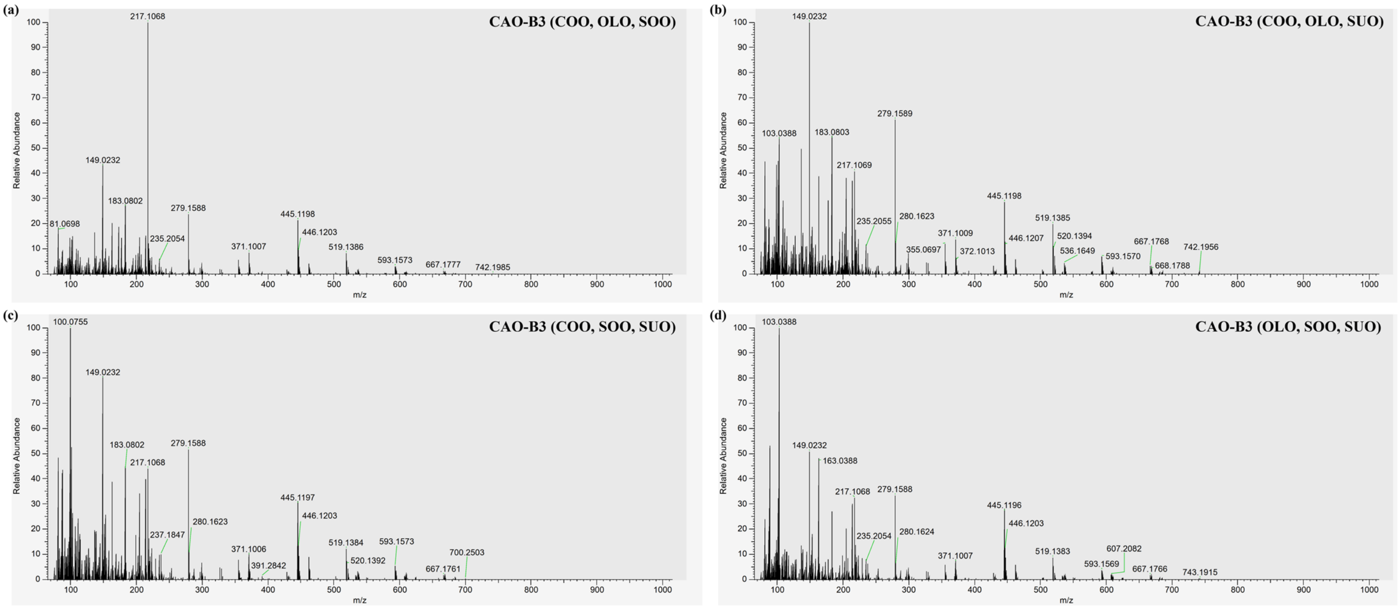

Figure S3 Representative mass spectra (V_B/V_A=50%: 50%) of quaternary adulteration system generated in positive ion mode by SICRIT-HRMS measurement.

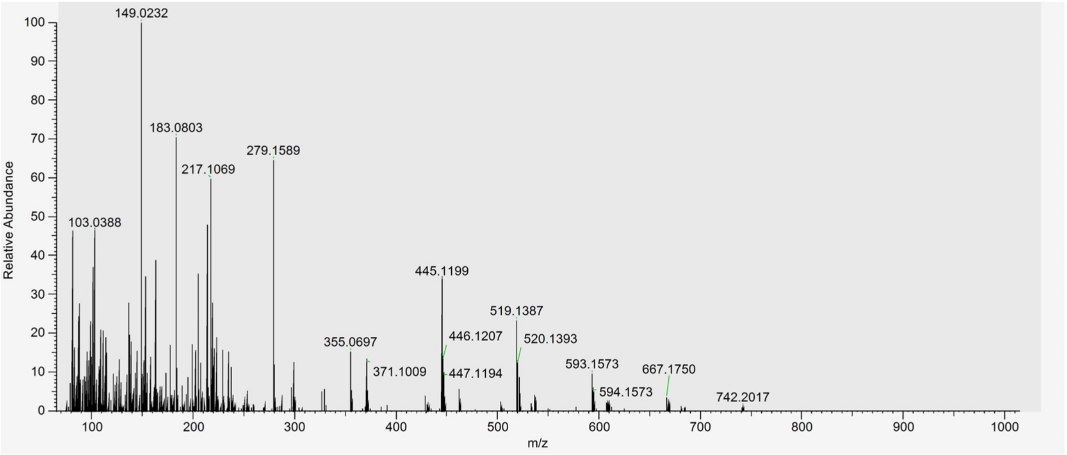

Figure S4 Representative mass spectra (V_B/V_A=50%: 50%) of quinary adulteration system generated in positive ion mode by SICRIT-HRMS measurement.
